# ASO Author Reflections: A Cost-Effectiveness Decision About Groin Lymphadenectomy: Reflections from the EAGLE FM Randomized Trial

**DOI:** 10.1245/s10434-025-17219-7

**Published:** 2025-03-23

**Authors:** Rashidul Alam Mahumud, Rachael Lisa Morton

**Affiliations:** 1 NHMRC Clinical Trials Centre, Faculty of Medicine and Health, The University of Sydney, Camperdown, NSW Australia; 2 Translational Research Hub, Poche Centre, Melanoma Institute Australia, The University of Sydney, Wollstonecraft, NSW Australia

## Past

The best surgical approach for treating metastatic melanoma involving groin lymph nodes has been a subject of considerable debate. Clinicians and researchers are split as to whether an inguinal lymphadenectomy (IL) or the more extensive ilio-inguinal lymphadenectomy (I-IL) is warranted. This controversy has persisted despite the evolving landscape of systemic therapies and evidence from trials such as MSLT-II1^1^ and DeCOG-SLT,^2^ which questioned the need for extensive nodal dissection after a positive sentinel node biopsy. However, definitive comparative data on these surgical approaches for patients with confirmed groin metastases were limited.

## Present

This economic evaluation alongside the EAGLE-FM trial (Mahumud et al.^3^) compared the costs and benefits of IL with I-IL for adults with stage III melanoma during 36 months. The results showed that IL was associated with improvements in quality-adjusted survival and reduced per-patient healthcare costs, predominantly due to shorter hospital stays. However, differences in costs, survival outcomes, and quality-adjusted life years (QALYs) did not reach statistical significance due to the limited sample size. The findings suggest that IL is a dominant surgical strategy, being overall slightly more effective and less expensive than I-IL. This underscores the importance of personalized, evidence-based treatment approaches, particularly for patients with limited access to essential cancer medicines and those unresponsive to immunotherapy or in settings wherein advanced first-line treatments are unavailable or prohibitively expensive.^2^

## Future

Future research could confirm these findings in larger studies with longer follow-up periods and examine the interplay between surgical management and systemic therapies. In addition, an economic evaluation that includes indirect costs and patient out-of-pocket expenses is warranted to fully capture the societal impact of these surgical interventions. Refining surgical protocols based on robust cost-effectiveness data will ultimately contribute to personalized, resource-efficient care for patients with metastatic melanoma. Given the growing availability of effective systemic therapies, de-implementation of I-IL surgery may be warranted to ensure that patients receive the most clinically and cost-effective surgical care.
